# Comparison of different treatments for children with radial neck fracture and analysis of prognostic factors

**DOI:** 10.1007/s00402-021-04178-3

**Published:** 2021-09-20

**Authors:** Anning Xia, Chao You, Jingming Han, Dechao Wu, Yongjie Xia, Jiangsheng Wang

**Affiliations:** Orthopaedics Department of Shenzhen Children’s Hospital, No. 7019, Yitian Road, Futian District, Shenzhen, Guangdong China

**Keywords:** Radial neck fracture, Children, Treatment, Open reduction, Close reduction

## Abstract

**Introduction:**

The aim of this was to analyze the effect of different treatment options on radial neck fractures in children and to explore the factors affecting the prognosis of fractures.

**Methods:**

The clinical data of 131 children with radial neck fractures admitted to our hospital from 2010 to 2018 were retrospectively analyzed, and the patients were divided into 6 groups according to treatment methods [manual reduction with Kirschner wires (K-wires) for internal fixation (group A); manual reduction with elastic stable intramedullary nails (ESINs) for internal fixation (group B); leverage reduction with K-wires for internal fixation (group C); leverage reduction with ESINs for internal fixation (group D); manual and leverage reduction with K-wires/ESINs for internal fixation (group E); and open reduction with K-wires/ESINs for internal fixation (group F)]. Postoperative elbow function and complications were analyzed.

**Results:**

Among the 131 patients with fractures, the median age was 8 years, the median preoperative angulation was 52°, the follow-up rate was 86.3% (113/131), the average follow-up time was 58.3 months, and the postoperative complication rate was 17.7% (20/113). The comparison among the different treatment groups showed that group B had the best recovery of elbow function, postoperatively, and the lowest postoperative complication rate. Age, duration of hospitalization, and preoperative angulation were independent factors affecting postoperative complications. Older age, longer duration of hospitalization, and higher angulation increase the postoperative complications.

**Conclusion:**

Different treatment options have different efficacies for radial neck fractures in children, of which manipulative reduction with internal fixation using ESINs can achieve good efficacy and a low postoperative complication rate. Age, duration of hospitalization, and preoperative angulation are independent factors for postoperative complications.

## Introduction

Radial neck fractures in children are an uncommon type of fracture, accounting for 1% of all body fractures and 5–10% of elbow fractures [1, 2]. Because multiple treatment options and differences among efficacy evaluations exist [1, 3–5], it may be difficult for some physicians to select a treatment approach. In addition, adverse outcomes are still reported in approximately 15–33% of such fractures [2] and can affect the lives of children. Therefore, by retrospectively analyzing the clinical data of 131 cases of radial neck fractures in children admitted to our hospital from 2010 to 2018, we explored the differences in the clinical characteristics of children treated with different surgical regimens and their efficacy, analyzed the factors influencing the efficacy of treatment for radial neck fractures in children, and summarized our experience to guide clinical work.

## Methods

**Fig. 1:**
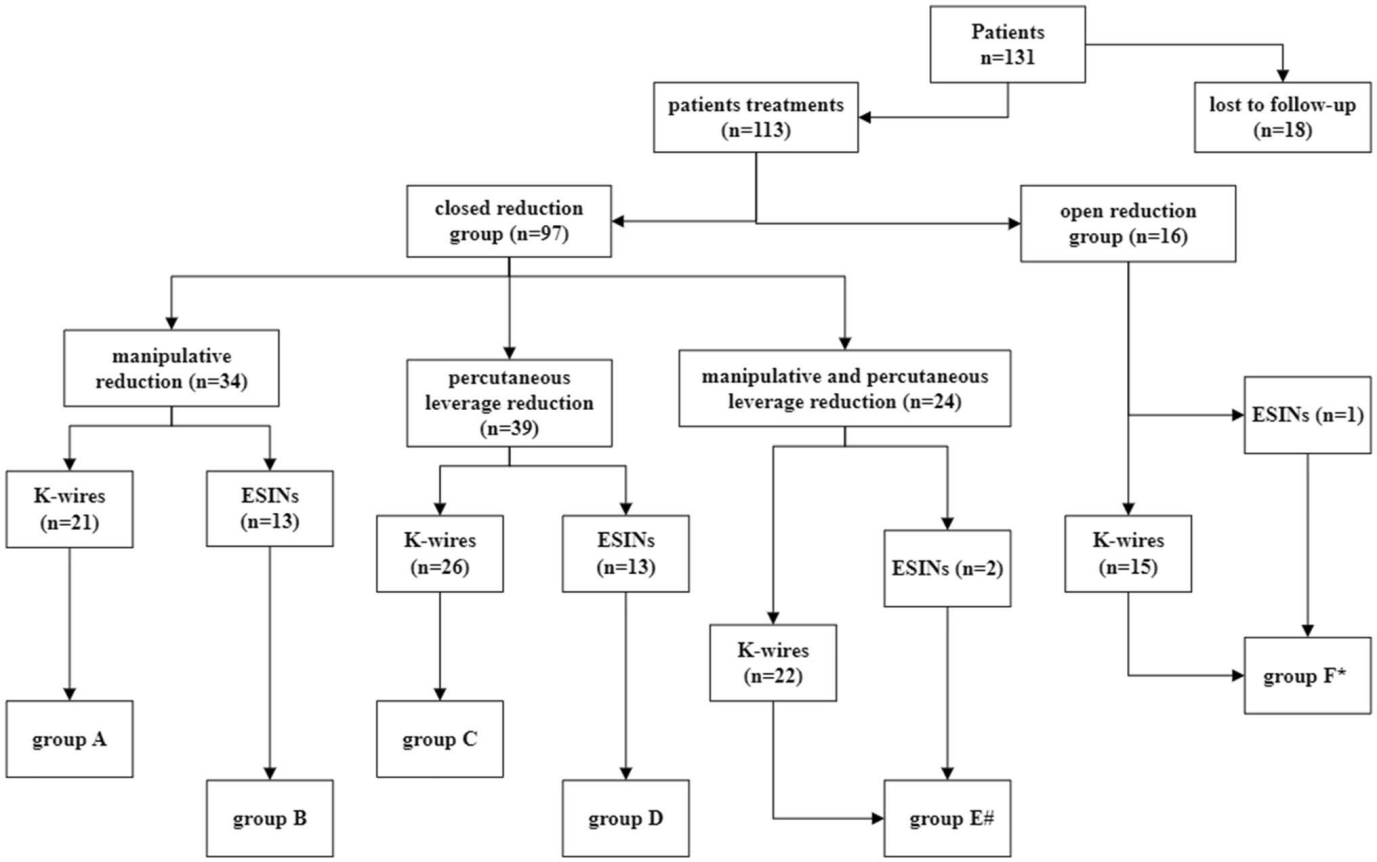
Grouping of patients. #:Because there was only 2 patients with manipulative and percutaneous leverage reduction with ESINs for internal fixation, the number of cases was small, and so, the 2 patients were included in group E. *: There was only 1 patient with open reduction with ESINs for internal fixation, so the patient was included in group F

All patient records in our hospital from January 2010 to December 2018 were retrospectively reviewed. The exclusion criteria were as follows: (1) multiple fractures, (2) conservative treatment, (3) nontraumatic radial neck fractures, and (4) accompanying medical diseases requiring special treatment, such as leukemia or malignant tumors. Preoperative fracture angulation and displacement were measured according to the Judet classification [6]. Postoperative complications included bony structural changes such as deformity of the radial head [7], premature physeal closure, osteonecrosis, heterotopic ossification, and stiffness, as well as neurovascular injury and postoperative nosocomial infection. The date of the last follow-up was October 31, 2020. Functional evaluations after treatment were conducted with the Tibone elbow outcome scoring system [8]. The patients were divided into six treatment groups according to surgical method and implantation material: group A, manipulative reduction with Kirschner wires (K-wires) for internal fixation; group B, manipulative reduction with elastic stable intramedullary nails (ESINs) for internal fixation; group C, percutaneous leverage reduction with K-wires for internal fixation; group D, percutaneous leverage reduction with ESINs for internal fixation; group E, manipulative and percutaneous leverage reduction with K-wires/ESINs for internal fixation; and group F, open reduction with K-wires/ESINs for internal fixation (Fig. 1). The 6 groups were divided into an open reduction group and a closed reduction group according to whether an incision of the brachioradial joint was needed to gain exposure during reduction. The patients were divided according to the different materials implanted during internal fixation into a K-wire treatment group and an ESIN treatment group for statistical analysis. Different treatment options in the closed reduction group were considered independently from each other, and when one of the closed reduction methods failed, open reduction surgery was performed. All patients received long arm casts for 3–8 weeks after surgery.

Manipulative reduction was performed mainly with the Patterson technique, Israeli technique, or Neher and Torch technique with pressure over the radial head and rotation of the forearm in various positions of elbow flexion and extension [9–11]. Percutaneous leverage reduction was achieved by inserting 1.5–2.5 mm K-wires into the fracture from the lateral side and elevating the wire proximally to reduce the displaced radial head. Open reduction was performed by making an incision using the Kocher approach. If fracture reduction was difficult during surgery, the annular ligament was incised to expose the fracture site and assist in reduction; then, the annular ligament was repaired after the end of reduction.

All 14 surgeons who participated in this study received standardized training in pediatric orthopedics, were certified, and had at least 5 years of working experience in pediatric orthopedics. All data were statistically analyzed using SPSS 20.0 software (IBM Corp, USA). The clinical data of the patients are described by statistical indicators such as the mean, standard deviation, median, interquartile range (IQR), and percentage. The Kruskal–Wallis, Chi-square, Mann–Whitney, Fisher’s exact, and Student’s t tests were used to statistically analyze the differences among different treatment groups. A logistic model was used to identify the prognostic factors affecting radial neck fracture results. All analyses were performed with two-sided tests and a test level *α* = 0.05, and *P* < 0.05 was considered to indicate statistical significance.

## Results

A total of 131 patients were included in this study, of whom 18 were lost to follow-up, with a median follow-up rate of 86.3% and a mean follow-up time of 58.3 months (range, 23–109 months). The median age of the patients was 8 years (IQR 6–10 years), and 45% were male. The median preoperative angulation in the 131 patients was 52° (IQR 39–65) (Table 1).

**Table 1:** Patient's clinical characteristics

		*n*
Male, *n* (%)	59 (45)	131
Age, median (IQR)–years	8 (6–10)	131
Duration of hospitalization^a^, median (IQR)–days	3 (3–4)	131
Weight, median (IQR)–-kg	26 (20.8–35)	131
Time of receive surgery^b^, median (IQR)–hours	15 (11–31)	131
< 48 h	13 (10–19)	108
≥ 48 h	96 (72–210)	23
Time of operation^c^, median (IQR)–minutes	60 (50–80)	131
Time of implant removal^d^, median (IQR)–days	38 (27.5–78)	113
Preoperative angulation, median (IQR)–-°	52 (39–65)	131
Cost of hospitalization^e^, median (IQR)–yuan	5532.2 (4485.4–7223.5)	131
Postoperative complication, *n* (%)		113
No	93 (82.3)	
Yes	20 (17.7)	
Judet classification, *n* (%)		131
II	12 (9.2)	
III	72 (55.0)	
IVa	30 (22.9)	
IVb	17 (13)	
Tibone elbow outcome scoring system, *n* (%)		113
Excellent	70 (61.9)	
Good	26 (23.0)	
Fair	11 (9.7)	
Poor	6 (5.3)	
Closed/open reduction groups, *n* (%)		131
Closed reduction	110 (84)	
Open reduction	21 (16)	
Implant materials, *n* (%)		131
Kirschner wire	102 (77.9)	
Elastic stable intramedullary nail	29 (22.1)	

^a^(Duration of hospitalization): total time spent on first hospitalization

^b^(Time of receive surgery): time interval from injury to start of surgical treatment

^c^(Time of operation): time spent on the first surgical treatment

^d^(Time of implant removal): the time interval between the first fixing of the implant and the last removal of it

^e^(Cost of hospitalization): all treatment costs for first hospitalization (RMB)

Of the 113 patients followed, there were statistically significant differences in age, duration of hospitalization, time of receive surgery, time of implant removal, preoperative angulation, Judet classification, hospitalization cost, and the Tibone elbow outcome score among the treatment groups (groups A–F). Among these factors, group B had the most “excellent” elbow scores (84.6%) without any “poor” scores, and group F had the minimal “excellent” elbow scores (43.8%) with most “poor” scores (18.8%). However, there was no significant difference in gender, weight, time of receive surgery, and postoperative complications among groups (Tables 2 and 3).

**Table 2:** Clinical characteristics of patients in different treatment groups

	*n* = 113	Group A, *n* = 21	Group B, *n* = 13	Group C, *n* = 26	Group D, *n* = 13	Group E, *n* = 24	Group F, *n* = 16
Male, n(%)	52 (46)	12 (57.1)	6 (46.2)	11 (42.3)	5 (38.5)	13 (54.2)	5 (31.2)
Age, median (IQR)–years	8 (6–10)	7 (5–9.5)	9 (7.5–11.5)	6.5 (4–9)	10 (7.5–11.5)	7.5 (6–10)	7 (6–10.8)
Duration of hospitalization, median(IQR)–days	3 (3–4)	3 (2–3.5)	3 (3–4)	3 (3–4)	4 (3–4.5)	4 (3–4.8)	5.5 (4–6)
Weight, median(IQR)–kg	26.2 (21.5–36.6)	26.2(20.5–35.8)	38 (23.7–41)	24 (19.5–27.8)	32.4 (24.7–40.1)	26.6(20.1–33.6)	25 (21.4–37.7)
Time of receive surgery, median(IQR)–hours	16 (11–31)	13 (9.5–22.5)	14 (11–17)	16 (11–32)	27 (10–60.5)	16 (11–34.5)	14 (11–90)
Time of operation, median(IQR)–minutes	60 (50–80)	50 (50–55)	60 (50–85)	60 (50–72.5)	60 (50–70)	60 (50–90)	90 (82.5–120)
Time of implant removal, median (IQR)–days	38 (27.5–78)	29 (25–39)	93 (83.5–137)	30 (27–37.3)	84 (70–102.5)	34.5 (27–41.8)	43 (28.3–65.3)
Preoperative angulation, median (IQR)–°	51 (37–62)	43 (28–59.5)	43 (32–57)	44.5 (32–58.5)	46 (38.5–76)	53.5 (45.3–67)	73.5 (60.5–90)
Cost of hospitalization, median (IQR)–yuan	5618.9(4584.0–11,025.6)	4338.3 (4083.4–5316.9)	12,414.2 (11,994.2–12,805.7)	4749.2 (4160.5–5566.7)	12,511.8 (11,799.9–13,452.8)	5144.5 (4557.1–6426.0)	6323.0 (6004.5–6949.5)
Postoperative complication, *n* (%)							
No	93 (82.3)	18 (85.7)	13 (100)	21 (80.8)	10 (76.9)	21 (87.5)	10 (62.5)
Yes	20 (17.7)	3 (14.3)	0 (0)	5 (19.2)	3 (23.1)	3 (12.5)	6 (37.5)
Judet classification, *n* (%)							
II	12 (10.6)	6 (28.6)	3 (23.1)	3 (11.5)	0 (0)	0 (0)	0 (0)
III	62 (54.9)	10 (47.6)	8 (61.5)	17 (65.4)	9 (69.2)	15 (62.5)	3 (18.8)
IVa	24 (21.2)	3 (14.3)	2 (15.4)	5 (19.2)	1 (7.7)	6 (25)	7 (43.8)
IVb	15 (13)	2 (9.5)	0 (0)	1 (3.8)	3 (23.1)	3 (12.5)	6 (37.5)
Tibone elbow outcome scoring system, *n* (%)							
Excellent	70 (61.9)	14 (66.7)	11 (84.6)	12 (46.2)	10 (76.9)	16 (66.7)	7 (43.8)
Good	26 (23.0)	6 (28.6)	2 (15.4)	9 (34.6)	1 (7.7)	5 (20.8)	3 (18.8)
Fair	11 (9.7)	1 (4.8)	0 (0)	3 (11.5)	2 (15.4)	2 (8.3)	3 (18.8)
Poor	6 (5.3)	0 (0)	0 (0)	2 (7.7)	0 (0)	1 (4.2)	3 (18.8)

Group A, manual reduction with Kirschner wires (K-wires) for internal fixation; Group B, manual reduction with elastic stable intramedullary nails (ESINs) for internal fixation; Group C, leverage reduction with K-wires for internal fixation; Group D, leverage reduction with ESINs for internal fixation; Group E, manual and leverage reduction with K-wires/ESINs for internal fixation; Group F, open reduction with K-wires/ESINs for internal fixation

**Table 3:** Comparison between different treatment groups

	*P* value
Gender	0.63
Age	0.009
C and D	0.049
C and B	0.024
Weight	0.102
Duration of hospitalization	0.002
F and A	0
F and C	0.018
Time of receive surgery	0.464
Time of operation	0
A and E	0.032
A and F	0
C and F	0
D and F	0.04
E and F	0.027
Time of implant removal	0
A and D	0
A and B	0
C and D	0
C and B	0
E and D	0.003
E and B	0
F and D	0.046
F and B	0
Judet classification	0
Preoperative angulation	0
B and F	0.002
A and F	0
C and F	0
Cost of hospitalization	0
A and F	0.001
A and B	0
A and D	0
C and F	0
C and B	0
C and D	0
E and B	0
E and D	0
B and D	0.041
Postoperative complication	0.16
Tibone elbow outcome scoring system	0.047

A group, manual reduction with K-wires for internal fixation; B group, manual reduction with ESINs for internal fixation; C group, leverage reduction with K-wires for internal fixation; D group, leverage reduction with ESINs for internal fixation; E group, manual and leverage reduction with K-wires/ESINs for internal fixation; F group, open reduction with K-wires/ESINs for internal fixation

In addition, the patients in the closed reduction group had relatively few postoperative complications [closed reduction group: (*n* = 14, 14.4%), open reduction group: (*n* = 6, 37.5%)] and relatively good Tibone function evaluation results [closed reduction group: excellent (n = 63, 64.9%), good (n = 23, 23.7%), fair (*n* = 8, 8.2%), poor (*n* = 3, 3.1%); open reduction group: excellent (*n* = 7, 43.8%), good (*n* = 3, 18.8%), fair (*n* = 3, 18.8%)]. However, the patients in the open reduction group had relatively large preoperative angulation degrees (mean in the closed reduction group: 49.6°, mean in the open reduction group: 73.5°, *P* < 0.05). The clinical data were compared between the K-wire group and the ESIN group, and significant differences were found in age (median in K-wire group: 7 years, median in the ESIN group: 10 years), weight (median in K-wire group: 25.0 kg, median in the ESIN group: 33.5 kg), time of implant removal (median in K-wire group: 32 d, median in the ESIN group: 93 d), and hospitalization cost (median in K-wire group: 5025.3 yuan, median in the ESIN group: 12511.8 yuan) (*P* < 0.05). Under the same conditions, ESINs were preferred for older patients.

There were 20 patients who presented 23 postoperative complications: 2 cases of nosocomial infection (acute upper respiratory tract infection), 5 cases of nerve injury (one of which was accompanied by radial head deformity), 1 case of osteonecrosis, 3 cases of premature physeal closure, 6 cases of radial head deformity [including one patient combined accidental injury (fell off the bed during hospitalization) and another case combined with elbow stiffness], 1 case of heterotopic ossification, and 2 cases of joint stiffness (Table 4). The logistic model showed that the treatment regimen was an independent factor affecting the Tibone elbow outcome score (*P* = 0.023, OR: 7.23, 95% CI: 1.318–39.67). Age (*P* = 0.009, OR: 0.71, 95% CI: 0.55–0.91), duration of hospitalization (*P* = 0.009, OR: 1.50, 95% CI: 1.11–0.02), and preoperative angulation (*P* = 0.018, OR: 1.04, 95% CI: 1.01–1.07) were independent factors affecting postoperative complications. The higher age, or increased preoperative angulation, or longer duration of hospitalization leads to higher rates of postoperative complications.

**Table 4:** Postoperative complications with different treatment options

	Group A	Group B	Group C	Group D	Group E	Group F
Radial head deformity	1^#^		2		2	1^*^
Nerve injury	2		1^★^	2		
Premature physeal closure						3
Joint stiffness				1		1
Nosocomial infection			2			
Heterotopic ossification						1
Osteonecrosis					1	

Group A, manual reduction with K-wires for internal fixation; Group B, manual reduction with ESINs for internal fixation; Group C, leverage reduction with K-wires for internal fixation; Group D, leverage reduction with ESINs for internal fixation; Group E, manual and leverage reduction with K-wires/ESINs for internal fixation; Group F, open reduction with K-wires/ESINs for internal fixation. #: The patient combined accidental injury (fell off the bed during hospitalization). ★: The patient was accompanied by radial head deformity. *: The patient combined with elbow stiffness

## Discussion

At present, there are many treatment options for radial neck fracture in children, and the reported conclusions differ [12–15]. A small number of patients have been included in the published papers, and the comparisons of different treatment options are relatively simple and cannot further explore the efficacy of different treatment options for children with radial neck fracture. For this reason, we retrospectively analyzed the clinical data of 131 cases, with outcome data available for 113 of radial neck fractures in children treated in our hospital during the past 8 years to compare the efficacies of different surgical treatment options and identify the factors affecting prognosis. In addition, to reduce interference from surgical schemes to treat multiple fractures [16–18], we specifically excluded such patients from the study.

Through statistical comparisons of the clinical data of patients who received different treatment regimens, we found that there was a statistically significant difference in age. Moreover, despite further statistical analysis, we failed to find a significant correlation between age and postoperative function, which is different from previous studies [17, 19, 20]. We consider that this difference may be related to factors such as different evaluation criteria for efficacy and different selection criteria for study subjects [1, 16]. Further high-quality studies are recommended to clarify this issue. Between the two groups stratified by internal fixation material, we found that older, heavier patients tended to receive ESINs for internal fixation. The hook at the front end of ESINs has a certain elasticity. ESINs can not only be fixed at the fracture site but also can rotationally reduce the fracture through the hook structure at the front end during the operation [4]. However, ESINs have a higher cost than K-wires, and the indwelling time in the body is longer.

As the blood supply to the radial head mainly passes through the reverse supply of the radial neck in children [2], it is currently considered that surgery should be performed as soon as possible to avoid further damage to the blood supply, but the specific time of receive surgery remains to be clarified [1, 21]. Therefore, this study analyzed the time from injury to received surgical treatment to explore whether this indicator has an effect on treatment efficacy. The statistical results showed that there was no significant difference in the time of receive surgery among different treatment groups (*P* = 0.464), and there was also no significant difference in the Tibone elbow outcome score and postoperative complications (*P* = 0.804 and *P* = 0.643, respectively); we did not find the time of receive surgery to be an independent predictor of efficacy, which was different from the results of previous studies [19, 21]. We speculate that this index (the time from injury to received surgical treatment) cannot completely reflect the radial head of blood supply injury and may be affected by whether adequate preoperative planning was achieved; moreover, we believe that premature surgical treatment may cause further injury, but the reasons need to be confirmed by further studies.

At present, there is no consensus on the application ranges of different surgical treatment options for preoperative angulation [17, 22–24]. In this study, it was found that there were differences in preoperative angulation among the groups, of which open reduction remained the main method for treating severe angulation (median: 73.5°, IQR 60.5–90°), but the postoperative complication rate was higher in this group (37.5%), and this group had the highest “poor” rate (18.8%) in the Tibone elbow outcome scoring system. Logistic model analysis showed that preoperative angulation was not an independent factor affecting efficacy and postoperative complications. Open reduction can fully expose the fracture site and allow for reduction under direct vision and repair of the damaged soft tissues. However, it is possible to cause further damage to the blood supply to the fracture site during the operation [25, 26]. There is no uniform conclusion regarding how many degrees of fracture angulation or how many centimeters of fracture displacement is necessary to recommend open reduction. Previous studies have shown that leverage reduction can be successful even in patients with a fracture angulation degree exceeding 60° or complete displacement [27]. Some scholars consider that if the preoperative imaging data suggest significant angulation and complete displacement, especially without contact with the metaphyseal margin, open surgical treatment can be used [26]. We believe that the degree of preoperative fracture angulation is not the only criterion for open reduction. Angles measured by preoperative radiography do not fully reflect the severity and reality of the fracture [28]. Our clinical experience suggests that open surgery may be required for patients with obvious swelling of the elbow, unstable dislocation of the elbow, or severe injuries (such as comminuted fracture or severe surrounding soft-tissue injury). When performing open reduction, we must try to protect the important surrounding soft tissues and blood vessels to avoid excessive separation of the soft tissues during the operation.

In this study, the Tibone elbow outcome scoring system was used to evaluate and compare the postoperative function of patients receiving different treatment regimens, and the results were statistically significant (*P* = 0.047). Overall, group B had the most excellent and good scores (excellent: 84.6%; good: 15.4%), because the effective manual reduction method together with the hook structure of the ESINs assisted in the reduction of the fracture, and the fracture site was firmly fixed with little damage to the surrounding soft tissues. These factors were conducive to repairing the fracture site, and good functional recovery was achieved. However, it should be noted that ESINs are not applicable to younger patients due to size limitations, and indwelling in the body for a longer time, and cost is higher. After comparing different treatment groups, we found no significant difference in postoperative complications (*P* = 0.164). The main evaluation indexes of the Tibone elbow outcome scoring system include patient symptoms (pain) and range of motion and carrying angle [8], but patients with abnormal imaging findings such as radial head deformity and premature physeal closure may have no limited motion or no pain symptoms [21, 25], which may explain the above results.

Age, duration of hospitalization, and preoperative angulation were independent risk factors for postoperative complications. The probability of postoperative complications was higher for patients with an older age, longer duration of hospitalization, and greater preoperative angulation. We inferred that these factors were associated with postoperative nosocomial infection. Previous studies have shown an association between the duration of hospitalization and postoperative nosocomial infection [29]. We found that children had a greater risk of postoperative nosocomial infection (mainly acute upper respiratory tract infection) with a longer duration of hospitalization, but a relatively low incidence of implant-caused infection. It is difficult to assess whether there is nerve injury (such as radial nerve injury) by examining the child’s finger movements in the early postoperative period due to factors such as pain, fear, or finger swelling. In this study, one patient who was found to have limited thumb movements on the 3rd day after the operation and was found to have postoperative radial nerve injury through examination received prompt treatment, and the recovery of this patient was good. However, with prolonged duration of hospitalization, which may facilitate early detection of nerve injury, the risk of nosocomial infection in children increases. In addition, according to our study findings, no patients had permanent nerve injury, and the vast majority of patients achieved complete repair approximately 2–6 months after surgery. Moreover, through long-term follow-up, we found that postoperative complications such as nosocomial infection had no effect on the patient's recovery of forearm movements. Based on the experience in our hospital, we recommend actively implementing enhanced recovery after surgery (ERAS) during the perioperative period [30], for example, oral and written information of patient and relative about all aspects of perioperative care, and the functional exercise of fingers was continued early after operation, and analgesics were given after operation. Manipulative reduction with ESIN fixation was the preferred surgical plan (incidence rate of postoperative complications: 0); with this approach, the duration of hospitalization can be shortened, the operational trauma is minimal, the fracture reduction effect is good, and the fracture fixation is reliable, all of which are conducive to postoperative functional recovery. Furthermore, parents are informed how to conduct finger movement examinations and functional exercises at home, to observe whether their children have limited finger movement and to contact doctors in a timely manner to assess whether there are complications such as nerve injury; this is conducive to the early detection of complications such as nerve injury and can allow for timely intervention to facilitate functional recovery in children.

This study is a single-center retrospective study and the research level is relatively low. However, based on the large number of samples from this clinical retrospective study, we can learn about the relevant clinical characteristics of radial neck fractures in children and also evaluate the efficacy of different treatment options, but further studies are required to confirm it.

## Conclusion

To obtain good functional recovery and a low incidence of postoperative complications, manual reduction combined with ESIN internal fixation may be the preferred treatment for radial neck fractures in children. However, for patients with large preoperative angulation degrees and severe injury, open reduction is still an indispensable approach. Performing reasonable and effective preoperative planning, shortening the duration of hospitalization, and applying other measures can reduce the occurrence of postoperative complications.
